# Diagnostic stability among chronic patients with functional psychoses: an epidemiological and clinical study

**DOI:** 10.1186/1471-244X-7-41

**Published:** 2007-08-16

**Authors:** Klaus D Jakobsen, Thomas Hansen, Thomas Werge

**Affiliations:** 1 Research Institute of Biological Psychiatry, Sct. Hans Hospital, DK-4000 Roskilde, Denmark; 2 University Department of Psychiatry, Psychiatric Centre Hvidovre, DK-2605 Broendby, Denmark; 3 Center for Pharmacogenomics, University of Copenhagen, 2200 Copenhagen N, Denmark

## Abstract

**Background:**

Diagnostic stability and illness course of chronic non-organic psychoses are complex phenomena and only few risk factors or predictors are known that can be used reliably. This study investigates the diagnostic stability during the entire course of illness in patients with non-organic psychoses and attempts to identify non-psychopathological risk factors or predictors.

**Method:**

100 patients with functional psychosis were initially characterised using the Operational Criteria Checklist for Psychotic Illness and Affective Illness (OPCRIT), medical records and health registers. To study the stability of diagnoses (i.e. shifts per time), we used registry data to define four measures of diagnostic variation that were subsequently examined in relation to four possible measures of time (i.e. observation periods or hospitalisation events). Afterwards, we identified putative co-variables and predictors of the best measures of diagnostic stability.

**Results:**

All four measures of diagnostic variation are very strongly associated with numbers-of-hospitalisations and less so with duration-of-illness, duration-of-hospitalisation and with year-of-first-admission. The four measures of diagnostic variation corrected for numbers-of-hospitalisations were therefore used to study the diagnostic stability. Conventional predictors of illness course – e.g. age-of-onset and premorbid-functioning – are not significantly associated with stability. Only somatic-comorbidity is significantly associated with two measures of stability, while family-history-of-psychiatric-illness and global-assessment-of-functioning (GAF) scale score show a trend. However, the traditional variables age-of-first-admission, civil-status, first-diagnosis-being-schizophrenia and somatic-comorbidity are able to explain two-fifth of the variation in numbers-of-hospitalisations.

**Conclusion:**

Diagnostic stability is closely linked with the contact between patient and the healthcare system. This could very likely be due to fluctuation of disease manifestation over time or presence of co-morbid psychiatric illness in combination with rigid diagnostic criteria that are unable to capture the multiple psychopathologies of the functional psychoses that results in differential diagnoses and therefore diagnostic instability. Not surprisingly, somatic-comorbidity was found to be a predictor of diagnostic variation thereby being a non-psychiatric confounder.

## Background

Some patients with functional psychoses experience a single diagnosis and few hospital admissions while others are subject to frequently changing diagnoses and repeated hospital admissions [1]. These differences may well reflect the underlying heterogeneity of the disorders that manifest themselves as a complex array of psychopathology and social features, which are often unique and individual. However, also factors of nosocomial nature – and thus unrelated to the illnesses themselves – may underlie the heterogeneous course of the diseases. Provokingly, only few factors of predictive value concerning the stability of diagnoses during the entire course of illness have been identified regarding functional psychoses e.g. schizophrenia, despite current focus on novel techniques like EEG or functional imaging attempting to measure physiological endophenotypes. The most reliable predictors seem to be age-at-onset and family-history-of-psychiatric-disease [2]. Diagnostic stability commonly refers to concordance/difference between a baseline and a follow-up examination and is used to study progression of the disease to identify prognostic factors of treatment responsiveness [3] or social function [4]. This perception of diagnostic stability does not embrace the many and diverse functional aspects or the diagnostic variation over the entire course of disease, incl. nosocomial factors. In order to study the stability of clinical diagnoses over the whole course of illness, we suggest a strategy that combines lifetime interview and clinical data with longitudinal information from the Danish health registers. We therefore hypothesize that an improved understanding of the diagnostic stability during the entire illness course of the functional psychoses, e.g. schizophrenia, may be better achieved by studying all available information on hospitalisation events between first and last diagnoses rather then the comparison of them.

First, we used lifetime-structured interviews (OPCRIT), medical records and the Danish health registers to collect putative factors affecting or predicting diagnostic stability. Second, we analysed these factors in relation to comprehensive data from the Danish health registers covering all hospital admissions of the particular subjects in order to their study impact on the diagnostic stability.

## Methods

### Ethics

The study has been carried out in accordance to the Helsinki Declaration. The Danish Data Protection Agency and the Danish Scientific-Ethical Committees (file # 01-024/01) have approved the study.

All patients had given written informed consent prior to inclusion into the project: Danish Psychiatric Biobank. At the time of recruitment and rating no subject was subdue to civil or forensic psychiatric restraint.

### Sampling and assessment

A cohort of 100 subjects with functional psychoses was randomly sampled among the 359 patients participating in the genetic research project, Danish Psychiatric Biobank, by 2004. The representative character of the examined sample is documented by the duration of illness (mean 20 yr.; 95%CI = 0–43 yr.) showing that both recent onset cases are represented as well as highly chronic patients [5]. Research assessment of the ICD-10 diagnoses were done using the OPCRIT instrument [6,7]. The results on quality of the clinical ICD-10 diagnoses and homogeneity i.e. reliability and agreement of diagnoses between the clinical and OPCRIT derived diagnoses have been described in detail elsewhere [5,8]. 79 had clinical and 74 had OPCRIT diagnoses of schizophrenia, where as 89 had clinical and 96 had OPCRIT diagnoses of schizophrenia-spectrum disorders, respectively. 2/3 of the OPCRIT interviews were done by a experienced PSE-certified consultant psychiatrist KDJ, who to avoid rater-drift lead an OPCRIT co-rating group with participating resident, research and consultant psychiatrists from the greater Copenhagen area during a two year period. A participating research resident psychiatrist JNF supervised by KDJ interviewed the remaining 1/3.

Further, clinical data, including the latest principal ICD-10 diagnoses, were extracted from interviews and medical records using a standardized schema made for the purpose with focus on the patient's history. [Generation of reliability measures regarding the clinical interviews using the standardized schema has not been possible due to the logistic set up of the study.] In order to improve the overall quality of the clinical data, all schemas on the 100 samples has been thoroughly proof read by consultant KDJ.

Information on all hospital contacts (incl. emergency room- and outpatient contacts, inpatient admissions and discharges) as well as corresponding principal ICD-8 diagnoses [1965–1994] and ICD-10 diagnoses [1994–2006] was obtained through the Danish, Psychiatric Central Research Register [9,9]. The Psychiatric Central Research Register contains complete electronic information on all psychiatric inpatient admissions since 1969 and on outpatients since 1995. Only inpatient status, which is always evaluated by a specialist in psychiatry, was used in this study. No private psychiatric hospitals exist in Denmark. Complete data on hospitalisations and diagnoses covering the entire duration of illness was available on 89 of the initial 100 subjects, leading to the exclusion of 11 patients. The basic clinical and epidemiological characteristics of these 89 subjects are shown in table 1.

**Table 1:** Basic demographic and clinical result on co-variables and putative predictors of diagnostic stability

**Putative co-variables of diagnostic stability**	**Value**	**Number^●^**	**25–75% Percentiles**
Alcohol or drug addiction ever present	Yes	70%	
Clinical Global Impression Scale (CGI)^▲^	5–7	50%	
Civil status, (i.e. ever married)	Yes	69%	
Employment at onset	Yes	66%	
Global Assessments of Functioning (GAF)^▲▲^	Score	40	(35–45)
Poor premorbid social or work adjustment	Yes	71%	
Somatic co-morbidity (any somatic illness ever present)	Yes	43%	
Treatment response on antipsychotics	Yes	95%	

**Putative predictors of diagnostic stability**			

Age of first admission	Year	25	(20–32)
Age of onset	Year	21	(17–30)
Duration of first admission	Days	37	(8–69)
Duration of hospitalisation first year of admission	Days	107	(50–295)
Family history of psychiatric disease	Yes	81%	
First diagnosis ICD-10 schizophrenia	Yes	23%	
First diagnosis ICD-10 schizophrenia spectrum disorder	Yes	53%	
Gender	Men	63%	
Sct. Louis Criteria for Schizophrenia^▲▲▲^	Yes	45%	
Year of birth	Year	1962	(1954–1970)

**Measures of hospitalisation events**			

Number of hospitalisations	(#)	14	(8–26)
Total duration of hospitalisation	Years	3,4	(1,7–6,0)
Total duration of illness	Years	17	(8–24)
Year of first admission	Year	1989	(1982–1999)

**Measures of diagnostic stability**			

Numbers-of-diagnostic-shifts	(#)	3	(1–6)
Numbers-of-diagnostic-spectrum-shifts^●^	(#)	1	(0–4)
Diagnostic-complexity (numbers-of-unique-diagnoses multiplied with numbers-of-diagnostic-shifts)^●●^	(#)	8	(2–22,5)
Spectrum-complexity (numbers-of-unique-spectrum-diagnoses multiplied with numbers-of-diagnostic-spectrum shifts)^●●●^	(#)	2	(0–9)

^●^Numbers of shifts between the three spectra assessed, i.e. schizophrenia spectrum, affective disorders and all others.

^●●^Diagnostic complexity was calculated as the sum of the numbers-of-unique-diagnoses multiplied with numbers-of-diagnostic-shifts ICD-8 and ICD-10, respectively.

^●●●^Spectrum complexity was calculated as numbers-of-unique-spectrum-diagnoses multiplied with numbers-of-diagnostic-spectrum-shifts for ICD-10 translated ICD-8 and ICD-10.

^●^Eleven of the 100 subjects in the sample were identified as having possible year of onset before 1969 (the year when the psychiatric register became electronic, no further information was available), these subjects were therefore excluded from analyses.

^▲^Guy, 1976; CGI scores were collapsed into the severe forms [score 5–7] vs. the milder forms [2-4].

^▲▲ ^Endicott et al, 1976.

^▲▲▲ ^Feighner et al, 1972.

ICD-8 (1965) was used as diagnostic criteria until 1994 as ICD-9 (1975) was newer introduced in Denmark. ICD-10 (1989) has been used from 1994 to present date as the diagnostic criteria. ICD-8 diagnoses relating to functional psychoses were translated into the corresponding ICD-10 diagnoses as shown in table 2 and described in detail by Ekholm et al [10].

**Table 2:** Diagnostic hierarchic clusters of register diagnoses

**Diagnostic cluster **	**ICD-8 diagnosis **	**ICD-9 diagnosis **	**ICD-10 diagnosis **
Schizophrenia	295.00, 295.10, 295.20, 295.30, 295.50, 295.60, 295.80, 295.99	295A, 295B, 295C, 295D, 295F, 295G, 295W, 295X	F20.0-3, F20.5-6, F20.9
Schizoaffective	295.70	295H	F25.0-2, F25.8-9
Schizophreniform	295.40	295E	F20.8
Psychosis NOS	298.00, 298.10, 298.20, 298.30, 298.99, 299.99	298A, 298B, 298C, 298E, 298W, 298X	F23.0-3, F23.8, F23.80-81, F23.9, F28.9, F29.9
Delusional disorder	297.00, 297.10, 297.98	297B, 297C, 297D, 297W, 297X	F22.0, F22.8, F22.9, F24.9
Bipolar	296.10, 296.20, 296.30, 296.88	296A, 296C, 296D, 296E, 296W	F30.0-2, F30.20-21, F30.8-9, F31.0-9
Depression	296.00	296B	F32.0-3, F32.30-31, F32.8-9, F33.0-4, F33.8-9
Other mental disorders	Any other diagnosis	Any other diagnosis	Any other diagnosis

Ekholm et al, 2005.

#### Definition of groups of variables

Three classes of variables were used:

##### Putative co-variables and predictors

A number of variables relating to events during illness course were obtained from the lifetime clinical & OPCRIT interviews. These variables primarily relate to the functional criteria of the St. Louis diagnostic system [11] as shown in table 1. Epidemiological variables associated with the healthcare system were obtained from the Psychiatric Central Research Register. These are shown in table 1 and include nosocomial markers, e.g. age-of-first-admission.

##### Measures of time

Four putative measures of time (i.e. observation periods or hospitalisation events) to be used in the analysis of stability were defined as shown in table 1.

##### Measures of diagnostic variation

Four complementary measures of diagnostic variation were defined (table 1): 1. Numbers-of-diagnostic-shifts. 2. Numbers-of-spectrum-shifts (i.e. shifts between the diagnostic spectra F2X, F3X and F-others). 3. Diagnostic-complexity, which was computed as numbers-of-diagnostic-shifts multiplied with numbers-of-unique-diagnoses (i.e. the number of distinct diagnoses used). For patients admitted prior to 1994, diagnostic-complexity was calculated separately for each diagnostic period (ICD-8: 1965–94; ICD-10: 1994–2006) and then summed. 4. Spectrum-complexity, which was calculated as numbers-of-spectrum-shifts multiplied with numbers-of-unique-spectra-diagnoses (i.e. the number of distinct spectra diagnoses used). The number of spectra is identical in the two diagnostic periods eliminating the need for separate counting in the ICD-8 and ICD-10 periods.

The two latter measures of diagnostic variation were created in order to discriminate repeated shifts that occur between the same few diagnoses from shifts between many diagnoses across the entire diagnostic systems during the course of illness.

### Data analyses and statistics

All data were administrated using the database MySQL^® ^and the scripting language PHP. All analyses were preformed using SAS^® ^9.1 and SigmaStat^® ^^3.1^. Univariant analysis was used to examine the relation between the four measures of diagnostic stability and the four measures of time. Bi-variate regression analyses were used to estimate the individual contribution to the diagnostic variation, while correcting for numbers-of-hospitalisations. Multiple regression analysis with backward elimination was used to determine which combination of independent variables that could explain the observed variation in numbers-of-hospitalisations.

## Results

Complete data on hospitalisations and diagnoses covering the entire duration of illness was available on 89 of the initial 100 subjects, leading to the exclusion of 11 patients. Data from the registers and clinical interviews revealed that these 89 subjects were highly phenotypic representatives of a cohort of chronic patients with functional psychoses, as shown in table 1. Two-thirds of the subjects were male and the age-of-onset and age-of-first-admission of the sample were identical to previous epidemiological findings. Most subjects had a family history of psychiatric disease, poor premorbid functional level or co-morbid addiction problems. The use of healthcare system related services showed a high frequency of hospitalisation, long-term admissions and long duration-of-illness, characteristic of severely ill subjects.

As preparatory step to study diagnostic stability, we first defined four different measures of diagnostic variation that describe the numbers of shifts in diagnoses and in diagnostic spectra as well as the complexity associated with these two type of shifts (for details see section on Methods). Table 1 shows that the two narrowly defined measures numbers-of-diagnostic-shifts and the derived diagnostic-complexity exhibit approximately twice the variation of the broader measures numbers-of-spectrum-diagnoses and the derived spectrum-complexity. This is not unexpected, as single diagnoses vary to greater extent then those of spectra.

Stability is defined as variation over time. As the next step we therefore attempted to identify the most appropriate measure of time with which to correct the measures of diagnostic variations (defined above) and thus allow us to study diagnostic stability. Consequently, four measures of time were defined (table 1): numbers-of-hospitalisations, total-duration-of-hospitalisation, total-duration-of-illness and year-of-first-admission.

Using univariant analyses we found that of the four time measures, number-of-hospitalisations, was significantly better than the other three time measures associated with all the four measures of diagnostic variation (table 3). Thus, numbers-of-hospitalisations was therefore used as an obligate variable as the best suited measure of observation period or hospitalisation events in the following analyses of diagnostic variation.

**Table 3:** Significant findings between the hospitalisation events and the stability measures. P-values and the corresponding R2-values given in parentheses

Measure of hospitalisation events*	Number-of- diagnostic-shifts*	Number-of- spectrum-shifts*	Diagnostic- complexity*	Spectrum- complexity*
Numbers-of-hospitalisations	0,0001 (0,501)	0,0001 (0,278)	0,0001 (0,421)	0,0001 (0,220)
Total-duration-of-illness	0,0005	0,02	0,0002	0,03
Year-of-first-admission	0,0006	0,03	0,0002	0,03
Total-duration-of-hospitalisation	0,09	0,2	0,07	0,3

* Univariant regression analyses using the stability measures as dependent variables and the hospitalisation events as the independent variables.

In order to study diagnostic stability, we performed bi-variate regression analyses with the four measures of diagnostic variation as dependent variables and numbers-of-hospitalisations as an obligate independent variable and each of the putative predictors or risk factors listed in table 1 as independent variables. Independent variables associated with one or more measures of stability after correction for numbers-of-hospitalisations can be said to be related to diagnostic stability.

We found that somatic-comorbidity was significantly correlated with the numbers of shifts in diagnoses and in spectra explaining approximately 3% of the diagnostic variation of each, but somatic-comorbidity was not associated to diagnostic-complexity or spectrum-complexity (see table 4, panel A). Family-history-of-psychiatric-disease and global-assessment-of-functioning (GAF) scale score showed a non-significant trend accounting for approximately 2% and 10% to the observed variation in numbers-of-diagnostic-shifts and diagnostic-complexity, respectively. Quite interestingly, no association was observed for age-of-onset and premorbid-functioning that are otherwise considered traditional markers of poor long-term outcome of illness.

**Table 4:** Significant findings between co-variables (upper part) and predictors (lower part) of diagnostic stability and the stability measures (panel A) and the number-of-hospitalisations (panel B). P-values are shown and the corresponding R2-values given in parentheses

	**Panel A**				**Panel B**
**Putative co-variables of diagnostic stability**^●◆^	**Number-of-diagnostic-shifts^●^**	**Number-of-spectrum-shifts^●^**	**Diagnostic-complexity^●^**	**Spectrum-complexity^●^**	**Number-of-hospitalisations^◆^**

Civil status, i.e. ever married					0,015
Global Assessments of Functioning (GAF)^▲^			0,069 (0,519)		
Somatic co-morbidity (ever present)	0,029 (0,528)	0,049 (0,309)			0,001

**Putative predictors of diagnostic stability^●◆^**					

Age of first admission					0,001
Family history of psychiatric disease	0,061 (0,522)				
First diagnosis ICD-10 schizophrenia					0,023
Sct. Louis Criteria for Schizophrenia^▲▲^				0,09 (0,246)	

^●^Bi-variant analyses using the four stability measures as dependent variables and numbers-of-hospitalisations in combination with each of the putative co-variables or predictors of diagnostic stability as independent variables.

^◆^Multiple regression with backward elimination using numbers-of-hospitalisations as the dependent variable and all the other co-variables and putative predictors as independent variables.

^▲^Endicott et al, 1976.

^▲▲ ^Feighner et al, 1972.

Numbers-of-hospitalisations explains as much as half of the observed diagnostic variation in this sample as shown in table 4, panel A. We therefore choose to examine, which combination of possible predictors or risk factors – if any – that predicts the variation in numbers-of-hospitalisations.

We analysed numbers-of-hospitalisations as the dependent variable in a multiple regression with stepwise backward elimination using the remaining co-variables and putative predictors listed in table 1 as independent variables. This analysis revealed that age-of-first-admission, civil status, first-diagnoses-being-schizophrenia and somatic-comorbidity are significantly associated to and combined explain two-fifth of the variation in numbers-of-hospitalisations (se table 4, panel B).

## Discussion

In this study we have addressed the intriguing feature that the illness courses of schizophrenia spectrum disorders are associated with considerable heterogeneity. One particular aspect of the heterogeneity is what appears as an inherent instability of the diagnoses of the individual when observed over the entire period of known disease. Three findings merit particular attention.

The first and foremost finding of our study is, somewhat to our surprise that the conventional markers of illness course examined in this study (i.e. age-of-onset and family-history-of-psychiatric-disease) have no significant explanatory power in relation to the four measures of diagnostic variation in this sample.

Second, among the examined independent variables, somatic-comorbidity significantly added explanatory power to two of the four measures of diagnostic variation (i.e. numbers-of-diagnostic-shifts and numbers-of-spectrum-shifts) but not to those of diagnostic-complexity or spectrum-complexity. Somatic-comorbidity is also highly significantly associated with numbers-of-hospitalisations (identified as the best measure of time in relation to stability; see below) and therefore explains the observed diagnostic variation in both an independently and dependently manner.

This is most likely because somatic-comorbidity in mentally ill patients raises the question of possible organic psychiatric illness when initially identified in the healthcare system. This leads to additional differential diagnoses, further diagnostic variation and more psychiatric hospitalisations due to possible liaison-psychiatric reasons. Once diagnosed, somatic-comorbidity becomes psychiatrically neutral, i.e. it does not cause the psychiatric diagnosis to change. However, it may cause more hospitalisations due to somatic admission during psychiatric admissions.

Third, the Finnish epidemiologists Isohanni and Moilanen [12,13] previously showed that the more severe illness and the less co-morbidity the more likely schizophrenia diagnoses are to be stable. Similarly, our data reveal discrete non-significant trends regarding family-history-of-psychiatric-disease and global-assessment-of-functioning (GAF) scale score that may or may not represent true associations that escape formal detection due to the size of sample.

Fourth, we find that numbers-of-hospitalisations is the best time measure to be used in relation to diagnostic stability, as it explains as much as half of the diagnostic variation. This is not surprising, as a contact with the healthcare system is necessary in order to have a diagnosis in the first place and to have following diagnostic shifts. We also find that the variation in numbers-of-hospitalisations itself can be significantly predicted by age-of-first-admission, civil-status, first-diagnoses-being-schizophrenia and somatic-comorbidity that account for up to two-fifth of the observed variation. This is anticipated, as subjects with an early onset of severe illness (e.g. schizophrenia) and somatic-comorbidity, who (additionally due to young age) are less likely to find a spouse, are more prone to experience additional contacts with the healthcare system.

Fifth, the diagnostic variation over time may equally well reflect the multiple variations in the psychopathology of the illnesses or presence of comorbid mental illnesses that at different hospitalisation events generates multiple differential diagnoses due to the present inflexible diagnostic criteria. The contemporary operational and categorical diagnostic systems are unable to embrace the whole phenomenology of the schizophrenia spectrum disorders, such as e.g. coexisting psychotic and affective symptoms, as previously described by our research group [8]. Our study also adds to the growing critique of the present clinical diagnostic systems due to problems with reliability and instable diagnoses regarding the functional psychoses e.g. schizophrenia, as also shown by Baca-Garcia et al [14] in their very recent and very large Spanish study. However, we cannot exclude that a fraction of the diagnostic stability is due to inappropriate use in the clinical settings of the current diagnostic criteria – despite that we only utilise diagnoses given or confirmed by specialists in psychiatry. Our study does not allow us to assess the extent of the clinical misdiagnosing or to decide on, which of the four measures of diagnostic variation to primarily use, as these equally seem to capture different aspects of the observed diagnostic instability.

Finally, our results demonstrates the utility of the Danish psychiatric registers to longitudinal validation of the stability of clinical diagnoses of patients that participate in research studies of e.g. genetic nature.

## Conclusion

This study presents a new experimental strategy to study diagnostic stability based on comprehensive data of the pattern of hospitalisation and diagnoses during the entire course of illness of the particular subject. This strategy identifies several risk factors or predictors of diagnostic instability in subjects with chronic schizophrenia spectrum disorders. The study also shows that none of the conventional predictors e.g. age-of-onset and family-history-of-psychiatric-disease is significantly associated with the diagnostic stability observed in this sample, whereas somatic-comorbidity is related to several measures of stability in both a direct and indirect manner. The study also revealed that the nosocomial variable, numbers-of-hospitalisations is the best measure of time in relation to diagnostic stability, and that the variation in numbers-of-hospitalisations itself may be explained by the variables age-of-first-admission, civil-status, first-diagnosis-being-schizophrenia and somatic-comorbidity.

## Competing interests

The author(s) declare that they have no competing interests.

## Authors' contributions

KDJ: Conception and design of the study and analysis, data analyses, interpretation of results and writing of the article.

TH: Design of data administration and analysis, data analyses, interpretation of results and reading, commenting and approval of the manuscript.

TW: Design of study and data analysis, interpretation of results, revision and final approval of the manuscript.

First and second author contributed equally to this work.

## Pre-publication history

The pre-publication history for this paper can be accessed here:


